# Coordination Compound (2,3,5-Triphenyltetrazolium)_2_[CuBr_4_] as Catalyst for the Curing Process of Epoxy Vinyl Ester Binders

**DOI:** 10.3390/ijms241411808

**Published:** 2023-07-22

**Authors:** Alexander E. Protsenko, Alexandra N. Protsenko, Olga G. Shakirova, Daria D. Zhelevskaya

**Affiliations:** 1 Department of Chemistry and Chemical Technologies, Komsomolsk-na-Amure State University, 681013 Komsomolsk-on-Amur, Russia; protsenko.chem@gmail.com (A.N.P.); shakirova_olga@mail.ru (O.G.S.);; 2 Institute of Automation and Control Processes, Far Eastern Branch, Russian Academy of Sciences, 690041 Vladivostok, Russia

**Keywords:** copper complexes, 2,3,5-triphenyltetrazolium, catalyst, curing, epoxy vinyl ester, activation energy, strength

## Abstract

This article presents a study on the synthesis and catalytic properties of copper complex (TPhTz)_2_[CuBr_4_] (here TPhTz is 2,3,5-triphenyltetrazolium). The obtained complex was characterized by various spectroscopic methods. The catalytic properties of the complex were evaluated in the curing of an epoxy vinyl ester system and their effectiveness was compared with that of cobalt octoate (its synonyms are known as Co(Oct)_2_, cobalt(II) 2-ethylhexanoate, cobalt isocaprylate, etc.). The catalyst was added at an amount of 2 w.%. The results showed that a 8 w.% solution of the complex provides catalytic properties with an activation energy of 54.7 kJ/mol, which is 25.2 kJ/mol higher than a standard curing system with Co(Oct)_2_. Thus, the solution of (TPhTz)_2_[CuBr_4_] in THF/DMSO accelerates the initiator decay process at room temperature, but for a longer time. The authors suggest that the curing mechanism may be accelerated by the appearance of (TPhTz)_2_[Cu^I^Br_3_] and free bromine in the system. A strength test of fiberglass-reinforced plastic revealed that the addition of this complex did not lead to a decrease in flexural strength and hardness. Thus, use of the complex allowed for the production of polymer composite products using vacuum-assisted resin transfer molding where an extended injection time was needed.

## 1. Introduction

Thermoset epoxies, polyesters, and epoxy vinyl ester (EVE) binders are commonly used in a wide range of industrial applications due to their excellent mechanical and chemical properties, thermal stability, and adhesion strength. These polymers are especially used as matrices for polymer composite materials such as fiberglass reinforced plastics (FGRP) [1,2,3] and carbon fiber reinforced plastics (CFRP) [4,5,6,7,8]. The automotive [9], marine [10,11,12], wind energy [13,14], aviation [15,16], and space [5] sectors are the major consumers of these materials.

There are two ways to obtain composite materials: autoclave [17,18,19,20] and out-of-autoclave [21,22,23] methods. Autoclaves are used for producing parts for aircraft industries, such as wings, blades, tail, and different panels [24,25,26]. In this case, thermal-cured resins are used [27]. For large objects such as ships, yachts, and wings for wind generators, out-of-autoclave methods are used. The vacuum assisted resin transfer method (VaRTM) is a great one-iteration way to manufacture massive objects [10,28]. To produce polymer composite materials using this method, it is necessary to use special binders [29,30]. They should have a low viscosity, which is necessary because composite parts are obtained by injecting the binder under vacuum into the filler located in a vacuum bag. Binders should also cure at room temperature and have a flexible gel time. At present, the gel time of most ester and epoxy vinyl ester systems ranges within an interval of 5–30 min. To increase the gel time, it is necessary to use special inhibitors, for example, 2,4-pentandion [31,32]. The recipe should also include an initiator and an accelerator (catalyst), which facilitates the decomposition of the initiator into radicals [33,34]. The inhibitor should be added to the binder in an amount of less than 1%. An error in the added mass can lead to too-long a binder curing or to a lack of curing. To solve this problem, it is proposed to use a less active accelerator. This approach can lead to an increase in the energy barrier of the curing reaction, resulting in a longer gel time. In addition, it is necessary to proceed with the reaction at room temperature.

Modern catalysts used as accelerators for epoxy vinyl ester and polyester binders are solutions of cobalt octoate or naphthenate [32,35]. In radical polymerization, catalysts are used to accelerate the reaction, control the molecular weight and molecular weight distribution of the polymer, and to improve the processability of the resulting polymer. Transition metal complexes can act as catalysts [36], generating free radicals through a redox process, or coordinating with a monomer or initiator to increase its reactivity [37]. Interest in such catalysts has grown over the years.

For example, it has been shown that tetrahalide complexes of copper(II) and cobalt(II) with N-heterocyclic compounds (L) of the general formula (HL)_n_[MHal_4_] (here, Hal is chlorine or bromine) catalyze various chemical reactions, including oxidation reactions, radical polymerization, the addition of hydrogen halides to unsaturated compounds, cyclization, and the metathesis of C–Cl bonds [38,39,40,41]. It was found that the tetrachlorocobaltate complex with a pyrimidine derivative is a catalyst for the oxidation of the catecholase enzyme under aerobic conditions [38]. Silva et al. [39] investigated the catalytic oxidation of cyclohexane under the action of hydrogen peroxide in the presence of a tetrachlorocuprate(II) complex with an organic base, bis(2-pyridylamine)amine. This complex exhibits high catalytic activity with a product yield of 68.9% of the total yield obtained within 24 h. Copper(II) coordination compounds, containing both the anion [CuCl_4_]^2−^ and a complex cation in which the ligand coordinates to Cu(II), catalyze the interaction between primary or secondary amines and aryl boric acid under mild conditions, with a reaction yield of over 96% [41]. Recently, we have shown the effect of (2,3,5-triphenyltetrazolium)_2_[CuCl_4_] on the destruction process of epoxy thermosetting plastic in a supercritical ethanol medium. The recycling process worked in pure ethanol at a minimum temperature of 250 °C, and it took 10 h to finish. At the temperature of 280 °C, the rate of the process increased. The fully recovered glass fiber was obtained after 4 h. The addition of 5% compound (2,3,5-TPhTz)_2_[CuCl_4_] of the solvent weight helped to reduce the process time to 2 h at a temperature of 250 °C [42].

The main purpose of this study is to investigate the catalytic activity and potential use of a new complex compound in the curing reaction of epoxy vinyl ester binders to produce large-sized polymer composite products with an extended injection time.

## 2. Results

### 2.1. Synthesis and Characterization

#### 2.1.1. Synthesis of Complex (TPhTz)_2_[Cu^II^Br_4_]

A solution of copper(II) oxide (0.2233 g, 0.001 mol) in concentrated hydrobromic acid (5 mL) was added to a solution of 2,3,5-triphenyltetrazolium chloride (0.3348 g, 0.001 mol) in water (10 mL) with a mole ratio Cu(II):TPhTz = 1:1. The resulting violet precipitate was filtered, washed with water and dried. The complex was obtained with a yield of 71%. Found: C, 47.8; H, 3.0; N, 11.7. Anal. Calcd. for C_38_H_30_Br_4_CuN_8_: C, 46.4; H, 3.0; N, 11.4.

Weighed portion (TPhTz)_2_[CuBr_4_] (0.5263 g) was dissolved in 10 mL of a mixture of dimethyl sulfoxide and tetrahydrofuran (1:1). The red single crystal (TPhTz)_2_[Cu^I^Br_3_] was obtained from the solution by slow crystallization for several weeks at room temperature.

#### 2.1.2. X-ray Structure Analysis

The crystallographic parameters of both (TPhTz)_2_[Cu^II^Br_4_] and (TPhTz)_2_[Cu^I^Br_3_] complexes are given in Table 1; the bond lengths and angles of complex anions are presented in Table 2.

#### 2.1.3. Fourier-Transform Infrared Spectroscopy

FTIR spectroscopic studies of the initial salt of 2,3,5-triphenyltetrazolium chloride, and the resulting complexes (TPhTz)_2_[CuBr_4_] and (TPhTz)_2_[CuBr_3_] were carried out (Table 3).

#### 2.1.4. Thermal Analysis

Thermal properties of complexes (TPhTz)_2_[CuBr_4_] and (TPhTz)_2_[CuBr_3_] were studied. Results are presented in Figure S4.

### 2.2. Catalytic Activity of Complexes in the Curing Reaction

The catalytic activity of the synthesized complex (TPhTz)_2_[CuBr_4_] was studied in the reaction of EVE binder curing with methyl ethyl ketone peroxide (MEKP) [33,43,44]. The coordination compound did not dissolve in the EVE. Since the heterogeneous catalysis is less effective than the homogeneous catalysis, the solubility of the compound was first investigated for the preparation of more preferred catalyst solutions.

#### 2.2.1. Solubility

The solubility of complex (TPhTz)_2_[CuBr_4_] was investigated to obtain a solution for the homogeneous catalysis of the decomposition reaction of methyl ethyl ketone peroxide during the curing of an EVE binder. A portion of the dried complex was added to solvents until the solubility limit was reached. The following solvents were tested: styrene, dimethyl sulfoxide (DMSO), tetrahydrofuran (THF), and dichloromethane (CH_2_Cl_2_). To determine the maximum solvent capacity, solutions of synthesized compound were prepared. The stability of the obtained solutions was also considered, which was determined by the presence or absence of sediment after 1 month. The resulting sustainable solutions and their concentrations are presented in Table 4. Solvent mixtures in a 1:1 ratio were also considered.

#### 2.2.2. Curing of EVE

The investigated binders consisted of three components: an epoxy vinyl ester binder, Derakane 411–350, which was accelerated with a catalyst and cured with methyl ethyl ketone peroxide. A solution of cobalt octoate (Co(Oct)_2_) was used as an accelerator in the curing reactions. The catalytic activity of the obtained complex (TPhTz)_2_[CuBr_4_] solutions was evaluated by adding them to the curing system to assess in gel and curing time. The catalytic activity was compared to the standard catalyst, Co(Oct)_2_, using a mixture with a catalyst content of 2 w.%, in accordance with the recipe provided in the methods. As the studied coordination compounds were dissolved in a different solvent to that of Co(Oct)_2_, the effect of the solvent on the gel time was also assessed. The solvent was added in an amount of 2 w.%. The experimental data are presented in Table 5.

#### 2.2.3. Hardness Test of Cured EVE

The hardness of the cured formulations was evaluated using the Shore D method to determine their strengths. The results are presented in Table 6.

#### 2.2.4. Thermal Analysis

**Figure 1:**
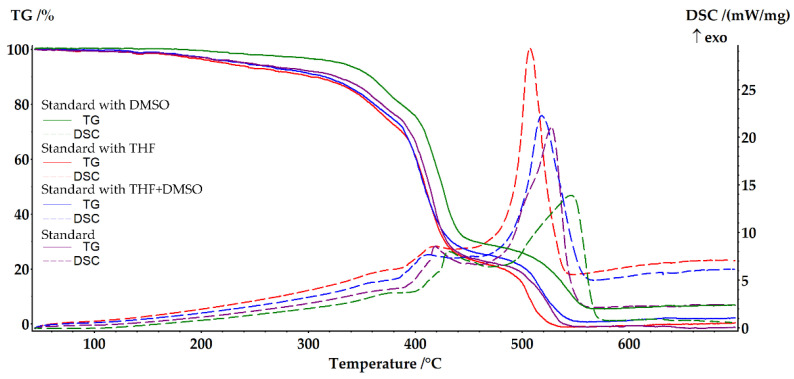
Thermal analysis of standard EVE with 2 w.% Co(Oct)_2_ with different solvents.

**Figure 2:**
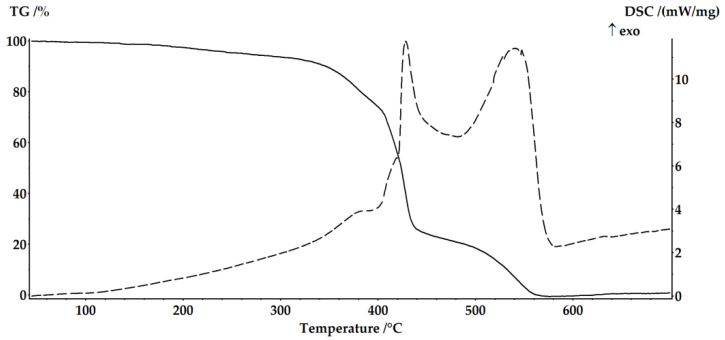
Thermal analysis of EVE with (TPhTz)_2_[CuBr_4_] in solution of DMSO/THF.

To investigate the thermal properties of the cured resin samples, measurements were conducted using a simultaneous thermal analysis device. The results of differential scanning calorimetry (DSC) and thermogravimetric (TG) analysis of selected cured samples, taken in heating mode from 40 to 700 °C with a heating rate of 10 K/min, are presented in Figure 1 and Figure 2.

**Figure 3:**
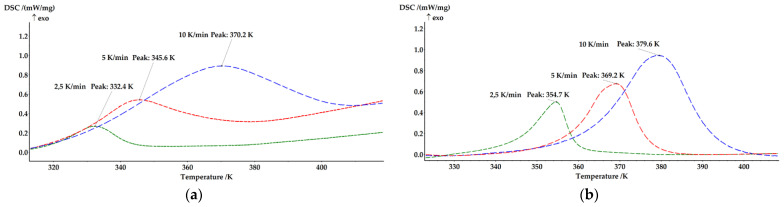

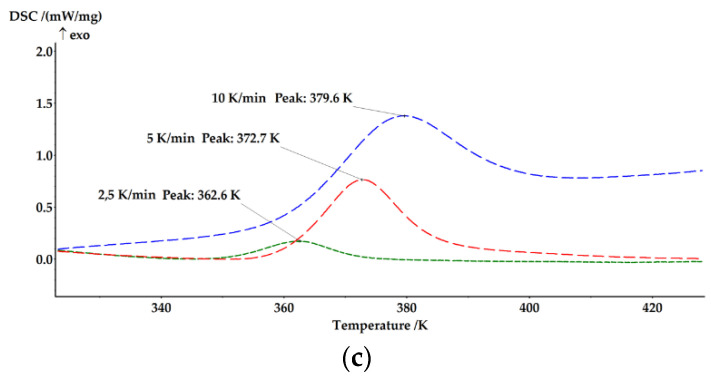
Thermograms obtained at speeds of 2.5, 5 and 10 K/min for: (**a**) Standard; (**b**) EVE with (TPhTz)_2_[CuBr_4_] and MEKP; (**c**) EVE with MEKP.

The curing process kinetics of systems containing the standard cobalt octoate catalyst, the (TPhTz)_2_[CuBr_4_] and only the initiator were studied to establish the catalytic effect of the complex. The analysis was conducted under non-isothermal heating using the Kissinger approach. The heating rates used were 2.5, 5, and 10 K/min. The results are presented in Figure 3.

#### 2.2.5. FTIR Spectroscopy

FTIR spectroscopic studies of the EVE binders cured in the presence of Co(Oct)_2_ and solution of (TPhTz)_2_[CuBr_4_] are shown in Table 7.

#### 2.2.6. Scanning Electron Microscopy

**Figure 4:**
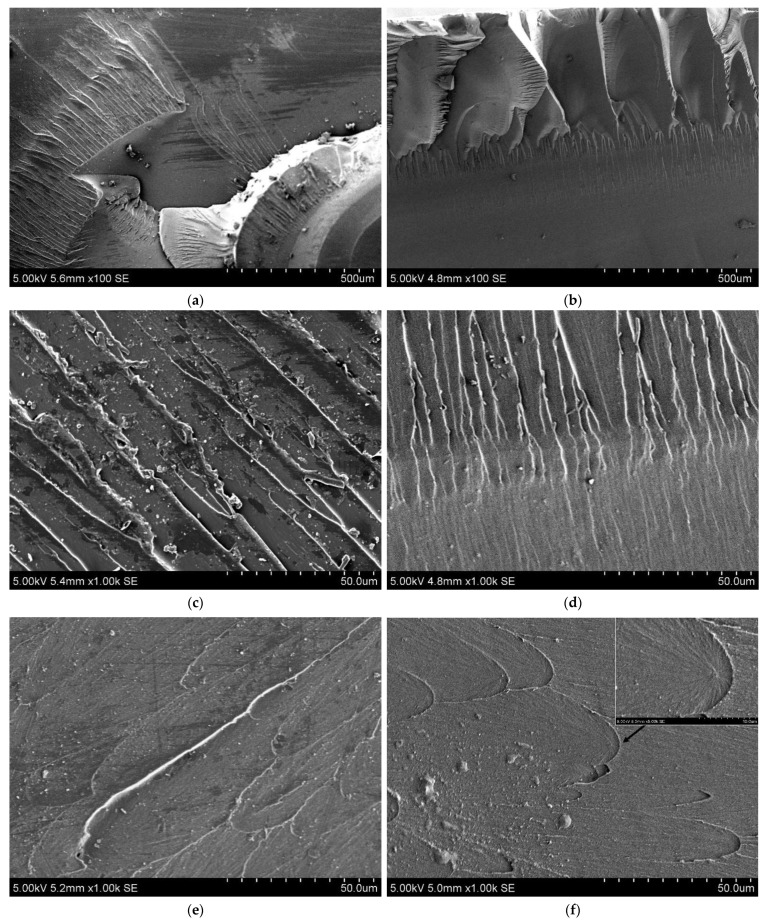
SEM images of fracture surface of the cured samples of EVE: (**a**,**c**,**e**) standard sample; (**b**,**d**,**f**) sample cured in the presence of complex (TPhTz)_2_[CuBr_4_].

Brittle chips of the cured samples of EVE were examined using the SEM method. Figure 4a,c,e shows fracture surfaces of a standard sample. Figure 4b,d,f shows fracture surfaces of a sample cured in the presence of complex (TPhTz)_2_[CuBr_4_] in DMSO/THF solution.

### 2.3. FGRP Production and Testing

#### 2.3.1. Production

**Figure 5:**
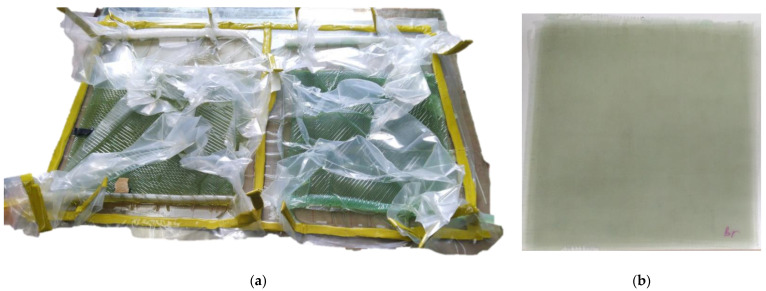
Photo: (**a**) VaRTM of the FGRP sample; (**b**) cured FGRP sample.

The epoxy vinyl ester binder was used with the investigated complexes and methyl ethyl ketone peroxide to produce FGRP using VaRTM (Figure 5). An FGRP sample made from the same batch of binder using a standard formula and cobalt octoate was used for comparison. The resulting plates were vacuumed until they cooled, then extracted and aged for one week under normal conditions. The FGRP was heated at 120 °C for 2 h to enhance its physical and mechanical properties, remove any remaining volatile components, and enable post-curing. The technological properties of the developed curing system were monitored during the production of FGRP, and it was noted that the lifetime was 1.5 h and the curing time was 3.5 h.

#### 2.3.2. FGRP Strength Test

Standard samples for testing for three-point bending were made from the obtained composites. The samples were examined before and after heat treatment to identify the effect of solvents on the strength of the material (Table 8).

## 3. Discussion

### 3.1. Synthesis and Characterization

#### 3.1.1. Synthesis

Complex (TPhTz)_2_[CuBr_4_] was obtained from an HBr acidified aqueous solution of copper(II) oxide with an ethanol solution of 2,3,5-triphenyltetrazolium chloride with Cu(II):TPhTz molar ratio 1:1. An elemental analysis of the compound showed that the complex has a composition with Cu(II):TPhTz molar ratio 1:2. The complex is highly soluble in alcohol, acetone, water and mineral acids. The compound does not dissolve in hydrocarbons.

When the complex (TPhTz)_2_[CuBr_4_] is dissolved in a mixture of dimethyl sulfoxide and tetrahydrofuran (1:1), copper(II) undergoes a reduction, resulting in the formation of a new copper(I) complex with composition (TPhTz)_2_[Cu^I^Br_3_]:

(TPhTz)_2_[Cu^II^Br_4_] → (TPhTz)_2_[Cu^I^Br_3_]↓ + Br^∙^

The authors suggest that the curing mechanism may be accelerated by the appearance of free bromine in the system, which is consistent with the findings of previous studies [45,46,47].

#### 3.1.2. X-ray Structure Analysis

**Figure 6:**
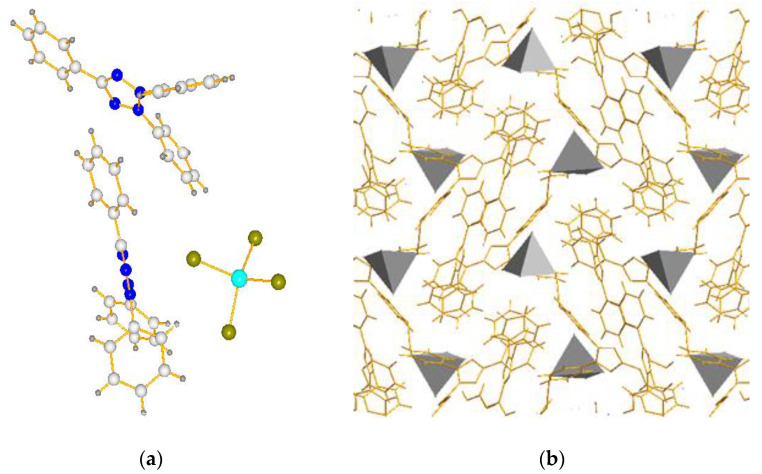
Crystal structure of complex (TPhTz)_2_[CuBr_4_]: (**a**) Unit; (**b**) Cell (C—white, N—blue, H—grey, Cu—sky blue, Br—yellow, tetrahedron - the coordination polyhedron of [CuBr_4_]^2−^).

**Figure 7:**
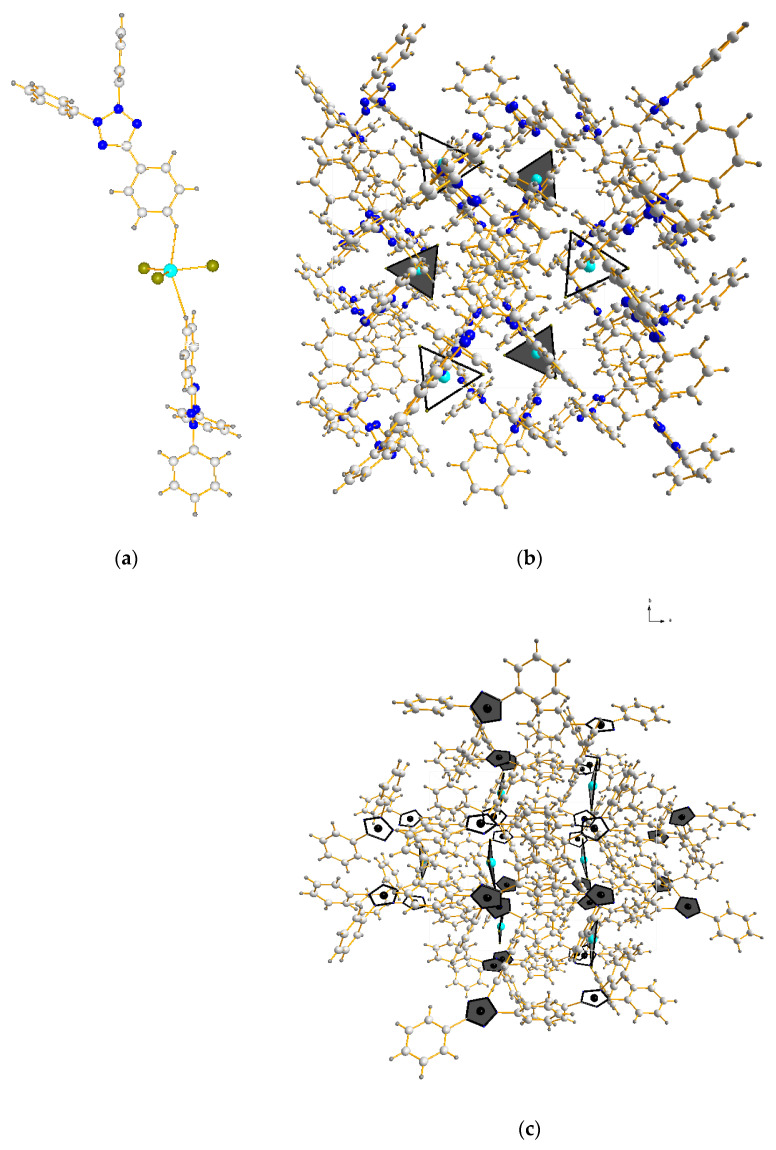
Crystal structure of complex (TPhTz)_2_[CuBr_3_]: (**a**) Unit; cell: (**b**) view 1, (**c**) view 2 (C—white, N—blue, H—grey, Cu—sky blue, Br—yellow, triangle- the coordination polyhedron of [CuBr_3_]^2−^, pentagon with the black center—tetrazolium π system.

X-ray diffraction analysis of complexes (TPhTz)_2_[CuBr_4_] and (TPhTz)_2_[CuBr_3_] (Table 1 and Table 2) indicates that both phases crystallize in monoclinic syngony. In the independent part, there are four formula units of the complex (Figure 6 and Figure 7).

In (TPhTz)_2_[CuBr_4_], the anion [CuBr_4_]^2−^ has the geometry of a distorted tetrahedron (Table 2). It is connected to the hydrogen atom of the meta-position of the 3-phenyl fragment of one of the organic cations by a short hydrogen contact Br…HPh (d_Br–C_ = 3.6355(1) Å). There are many examples of weaker interactions in the literature (d_Br–C_ = 3.7–3.9 Å) [48,49,50]. The crystal packing of (TPhTz)_2_[CuBr_4_] is formed due to the arrangement of inorganic anions in a staggered order with their edges to each other, and organic cations are located between them (Figure 6).

Analysis of the isolated single crystal of (TPhTz)_2_[CuBr_3_] shows that the anion [Cu^I^Br_3_]^2−^ has the shape of a flat triangle with different angles Br-Cu-Br (Table 2). The coordination polyhedron of copper(I) is completed to a trigonal bipyramid due to the hydrogen atoms of the para-position of the 5-phenyl fragment of two organic cations. In the crystal of the complex (TPhTz)_2_[CuBr_3_], the triangular fragments of the [Cu^I^Br_3_]^2−^ anion line up perpendicular to the tetrazole cycles of cations (Figure 7c).

When comparing the geometric parameters, there are no significant differences in the Cu–Br bond lengths for both complexes, but there is a significant difference in the angles and shapes of the coordination polyhedra characteristic of the corresponding valences of the central metal-complexing agent.

#### 3.1.3. FTIR Spectroscopy

In the high-frequency region of the IR spectra of the initial salt TPhTzCl and complex (TPhTz)_2_[CuBr_4_] and (TPhTz)_2_[CuBr_3_], the bands of stretching vibrations of phenyl rings are present in the 1600 cm^−1^ region. Bands of stretching vibrations of the tetrazole ring are observed at 1527–1528 cm^−1^. The intensity and position of these bands do not differ significantly, which indicates that the structure of the initial cation (TPhTz)^+^ remains unchanged in the resulting coordination compound.

#### 3.1.4. Thermal Analysis

In accordance with the TG data, in air, the complex (TPhTz)_2_[CuBr_4_] is stable up to 285.5 °C. The (TPhTz)_2_[CuBr_3_] loses 4.18% of mass at the temperature 140 °C, which is occurred with the evaporation of DMSO. After that, the compound is stable up to 230 °C. DSC revealed the existence of reversible phase transitions in both complexes, corresponding to the process of melting. This point registered at the temperature of 197.8 °C for (TPhTz)_2_[CuBr_3_] and at the temperature of 187.4 °C for (TPhTz)_2_[CuBr_4_].

### 3.2. Catalytic Activity

#### 3.2.1. Curing of EVE

As a result of the performed studies, it was established in the standard recipe that the binder gel time is 16 min (Table 5). This time is acceptable to inject the binder in the composite parts with the area of 2–4 m^2^, depending on the type of filler, configuration of the product and scheme of the injection points. To produce large constructions such as ships, airplane parts and wind blades could take much more time. Different catalytic systems were researched to prepare a composition with a gel time of about 1 h. The addition of pure co-solvents from the considered series leads to an increase in gel and curing time. The addition of THF influences the curing process to a lesser extent, and this inhibition effect is not sufficient to achieve the desired goal. The use of a solvent mixture in a system containing 2% of Co(Oct)_2_ has less effect on the life of the binder but increases the curing time by almost two times. The addition of a mixture of DMSO and THF solvents to the binder has less of an effect on the curing process. According to the data presented in Table 5, the gel time is increased by 3 min, and the curing time is increased by 9 min. The remaining mixtures increase at least one of the parameters to a greater extent. The increase in gel time is apparently due to the need to remove the solvent from the cured binder and its compatibility with it.

Depending on the combination of the solvent with the complex, the curing parameters significantly vary. At the same time, the THF/DMSO system has a minimum gel time that is two times higher than that of the standard formulation. The maximum lifetime was achieved by introducing a solution of the complex in styrene. The lifetime was approximately 5 h, and the total curing time exceeded 10 h.

Among the results obtained to create a curing system for large-sized structures with a long injection time, catalysts that provide a gel time of approximately 1 h are of the greatest interest. These include 8 w.% of catalyst (TPhTz)_2_[CuBr_4_] in a solution of THF/DMSO and 6 w.% of catalyst (TPhTz)_2_[CuBr_4_] in a THF solution.

#### 3.2.2. Hardness Test of Cured EVE

To assess the strength properties of the cured formulations, the samples’ hardness was determined by the Shore D method. The results are presented in Table 6.

Based on the results presented in Table 6, the recipe was selected with a hardness that was not lower than that of the comparison sample. The hardness was evaluated both before and after heat treatment. Further investigations were carried out using solution (TPhTz)_2_[CuBr_4_] 8 w.% in a THF/DMSO, which exhibited a hardness of 83 after curing that was similar to the reference sample. After heat treatment at the temperature of 120 °C for 2 h, the hardness increased to 88, which is two units higher than that of the sample based on cobalt octoate. The sample of the binder cured with a solution of (TPhTz)_2_[CuBr_4_] in THF has lower hardness in both states, both before and after heat treatment.

#### 3.2.3. Thermal Analysis

The Figure 1 displays thermograms of cured epoxy vinyl ester resin samples in the presence of cobalt octoate catalyst and different solvents. All samples were treated at a temperature of 120 °C for 2 h after curing. The impact of the introduced solvents on the thermal properties of the polymers is also shown. All samples behave similarly up to 120 °C, indicating the removal of all volatile components during post-curing. At temperatures above 120 °C, a small mass drop is observed without a visible response to the TG curve, indicating the removal of unbound styrene residues. Above 160 °C, the destruction process starts, accompanied by an exothermic peak in the DSC curves, and the mass loss steps are identical for all samples.

According to a thermal analysis of EVE with (TPhTz)_2_[CuBr_4_] in a solution of DMSO/THF (Figure 2), using a complex compound solution instead of cobalt octoate produces a polymer with the same operating temperature range and identical mass drop curves, but the enthalpy of the main stages of the destruction process differs by 0.87 kJ according to DSC data (Table 9).

The curing process activation energy of the new binding systems was determined by the Kissinger method. The maximum temperatures of the curing peaks were identified. Values of ln(v/T^2^) and 1000/T were calculated and are presented in Table 10.

**Figure 8:**
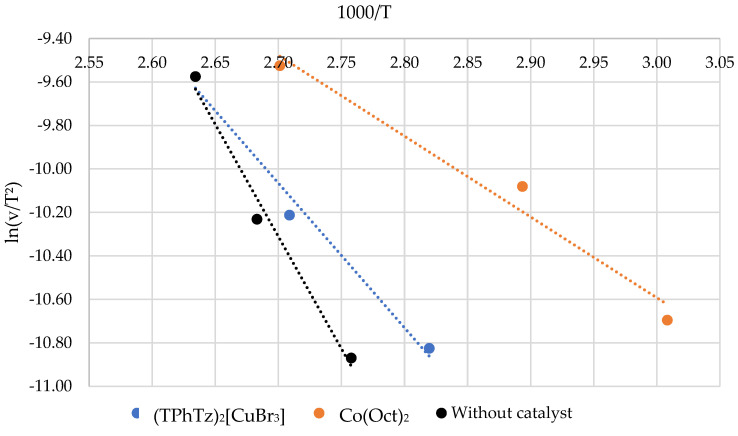
Kinetic Kissinger curves.

The data obtained from Table 10 were used to construct the kinetic curves shown at Figure 8 The activation energy (Ea) was then determined using the equation Ea = -R·tg(α), where tg(α) represents the tangent of the slope angle of the curve, and R is the universal gas constant, which is equal to 8.314 J/(K·mol). The Ea values calculated using the Kissinger approach are presented in Table 11.

The obtained results confirm that an 8 w.% solution of (TPhTz)_2_[CuBr_4_] functions as a catalyst for the curing of the EVE binder. The activation energy for this system was determined to be 54.7 kJ/mol, which is significantly lower than that of the EVE/MEKP system by 30.6 kJ/mol. Additionally, the activation energy of the curing reaction in the presence of Co(Oct)_2_ was found to be 23.7 kJ/mol lower than that of the system investigated in this study. The experimental data obtained for the activation energies are consistent with the literature values [34,51,52]. This can lead to a longer gel and curing time in the presence of complex (TPhTz)_2_[CuBr_4_] compared with Co(Oct)_2_.

#### 3.2.4. FTIR Spectroscopy

Due to the overlapping of the oscillation bands of the catalyst complex with the organic polymer matrix, it is impossible to speculate about any structural changes in the (TPhTz)^+^ fragment. However, the authors suggest the possibility of changes in the tetrazole fragment, such as cycle opening or copolymerization, which are not detectable in the sample due to their low amount. The IR spectra of the resins show characteristic bands of valence vibrations, such as (OH, CH_3_, CH_2_, COO) and R(benzene) (Table 7).

The oscillation band at 3444 cm^−1^ is related to hydroxyl groups of the epichlorohydrin, while the stretching vibrations due to carbonyl groups in methacrylate are observed at 1725, 1716, 1710, and 1181 cm^−1^ [53,54,55].

Despite the use of different solvents and catalysts, the IR spectroscopic analysis of the cured resins indicates that there were no significant changes in their qualitative composition. Furthermore, there were no observed S=O fluctuations in the sulfoxide group at 1225–980 cm^−1^, but there was vibration –S–O– at 670 cm^−1^_,_ which suggests that DMSO was involved in copolymerization.

#### 3.2.5. Scanning Electron Microscopy

The fracture surfaces of the cured binders from the standard system and samples obtained in the presence of (TPhTz)_2_[CuBr_4_] solution were found to be very similar. Figure 4a,b show the general view of the fracture surfaces. Mirror zones, transition zones, and relief zones are highlighted [56,57,58,59]. In both cases, river-like lines were observed (Figure 4c,d). However, there is a difference between the fracture surfacesL the number of fine river markings appear to be somewhat greater for the modified sample. In addition, Figure 4d, showing the fractograms, shows that the sample cured in the presence of the complex has more branches and a smaller object height compared to the standard sample. The standard sample exhibits river-lines with a larger height and more twisting due to its higher plasticity [58,60]. On the other hand, the fracture surface of the sample cured in the presence of the (TPhTz)_2_[CuBr_4_] complex (Figure 4f) displays diverging branches of parabolas and a lower number of structural objects, indicating a different fracture pattern. Figure 4e shows that the structural objects were observed to be much larger. The parabolas in the fracture surface are attributed to the presence of micropores, which is shown in the high-magnification images. The sample cured in the presence of (TPhTz)_2_[CuBr_4_] solution shows a smaller number of microdefects, which is most likely due to the longer gel time, resulting in more complete degassing of the binder. EDX analysis at the mirror zone demonstrates a homogeneous distribution of copper (8.05 keV), bromine (11.92 keV), and sulphur (2.31 keV) in a cured EVE binder (Figure S8).

### 3.3. FGRP Strength Test

A slight difference was found in the Shore D hardness between the samples obtained using the standard curing system and those containing (TPhTz)_2_[CuBr_4_] as a catalyst. However, the composites still exhibited higher hardness than the cured polymers listed in the table due to reinforcement.

After heat treatment at 120 °C for 2 h, flexural strength increased by 38%, which is attributed to the removal of residual volatile components, particularly non-reacted styrene [61,62,63]. The difference in the strength values among the cured samples was minor, with a 3 MPa increase in strength for the standard sample, which represents only 0.7% of its total strength.

The addition of (TPhTz)_2_[CuBr_4_] solution did not prevent decreases in the flexural strength and hardness of the cured resin. That is why it became possible to use in industrial applications to produce large polymer composite products with an extended injection time.

## 4. Materials and Methods

**Figure 9:**
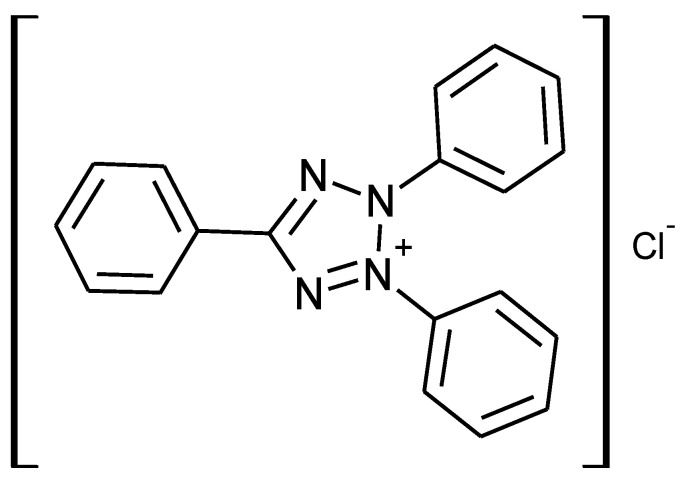
2,3,5-triphenyltetrazolium chloride (TPhTzCl).

The researched binders were composed of three components: 100 w.% of Derakane momentum 411–350 (Ashland, Wilmington, DE, USA), 2 w.% of catalyst, and 2 w.% of Butanox M-50 (methyl ethyl ketone peroxide). A solution of 1% cobalt octoate (NL-49P Akzo Nobel, Amsterdam, The Netherlands) was used as an accelerator in the curing reactions of epoxy vinyl ester binders using MEKP. The researched catalyst used was a copper(II) bromide complex with organic heterocyclic nitrogen-containing cations. For its synthesis, high-purity grade CuO, 2,3,5-triphenyltetrazolium chloride (TPhTzCl) (as shown in Figure 9) and concentrated HBr acid were used.

Elemental analysis of the C,H,N of the compound was carried out by the elemental analyzer EURO EA 3000 produced by EuroVector (Pavia, Italy).

Infrared spectra (4400–4000 cm^−1^) were recorded on an IRAffinity-1S FTIR spectrometer (Shimadzu, Kyoto, Japan) with 0.5 cm^−1^ resolution and 30,000:1 signal-to-noise ratio (peak to peak) in KBr pellets.

X-ray powder diffraction analysis was carried out on a Bruker–Nonius X8Apex automatic four-circle diffractometer equipped with a two-coordinate CCD detector at temperatures of 150(2) K or (296(2) K), with the help of radiation from a molybdenum anode (λ = 0.71073 Å) and a graphite monochromator. Absorption was empirically accounted for due to the SADABS program (version 2008/1) [64]. The structure was completed using the direct method and refined by full-matrix least squares in an anisotropic approximation for nonhydrogen atoms using the SHELXTL package [65]. Hydrogen atoms for organic ligands were refined using rigid body approximation.

The study of catalytic activity was carried out by introducing solutions of complex compounds into the binder in the amount of 2 w.%. The gel time of researched binders was determined with ISO 2535:2001 [66]. According to the method, 50 g of the oligomer at 25 °C was added to the polypropylene glass. Next, the researched catalyst was added and mixed. After that, the peroxide was added and carefully mixed. The prepared binder was termostated at a temperature of 25 °C. The consistency of the binder was checked with a spatula. Time was stopped when the binder stopped draining from the spatula. Also, the curing time was recorded as the moment at which the binder cooled down and lost its stickiness.

A thermal analysis of the samples was performed on an STA 409 PC Luxx simultaneous thermal analyzer manufactured by NETZSCH-Gerätebau GmbH. Thermogravimetric (TG) and differential scanning calorimetric (DSC) data were recorded during the experiment. The analysis was carried out in corundum ceramic crucibles. The heating was carried out at the rate of 10 K/min in the air. A study of the kinetics used to cure the binder with various catalysts was carried out using the Kissinger method [67,68]. In this case, the experiment was carried out at three different rates of 2.5, 5 and 10 K/min.

The hardness of the systems cured in the presence of various catalysts was evaluated using a HARDNESS tester with the D scale according to ISO 868:2003 [69].

Microstructural studies were carried out using a Hitachi S-3400N scanning electron microscope (SEM) with a tungsten cathode electron gun. The measurements were carried out at an accelerating voltage of 5 kV using a secondary electron detector (SE).

## 5. Conclusions

Copper(II) complex with 2,3,5-triphenyltetrazolium chloride in an HBr acidic medium of composition (TPhTz)_2_[CuBr_4_] was synthesized. X-ray diffraction data showed that the anion [CuBr_4_]^2−^ is a distorted tetrahedron.

To investigate the catalytic properties of complex (TPhTz)_2_[CuBr_4_], the possibility of its introduction into the reaction system was studied. The coordination compound did not dissolve in the EVE. Since heterogeneous catalysis is less effective than homogeneous catalysis, the solubility of the compound was investigated. Several solvents were examined, including styrene, dimethyl sulfoxide, tetrahydrofuran, and dichloromethane, as well as solvent mixtures in a 1:1 ratio. The findings revealed that the presence of DMSO resulted in the reduction from Cu(II) to Cu(I) and the formation of the (TPhTz)_2_[CuBr_3_] compound. The anion [CuBr_3_]^2−^ has the shape of a flat triangle. The geometric parameters of complex (TPhTz)_2_[CuBr_3_] do not differ significantly from the parameters of compound (TPhTz)_2_[CuBr_4_]. There is a significant difference in the angles and shapes of the coordination polyhedral characteristic of the corresponding valences of the central metal-complexing agent.

The catalytic activity of designed systems with complex (TPhTz)_2_[CuBr_4_] was compared to the standard catalyst for curing process, Co(Oct)_2_. The catalyst was added at an amount of 2 w.%. The use of a catalyst solution that provides a gel time of 1 h was based on the results of modeling the impregnation of large-sized structures. This solution contained 8 w.% of the catalyst (TPhTz)_2_[CuBr_4_] in a mixture of THF/DMSO. The binder cured in the presence of this solution exhibited a Shore D hardness of 83. After heat treatment at a temperature of 120 °C for 2 h, the Shore D hardness increased to 88.

Data from thermal and FITR analyses show that samples cured in the presence of catalyst (TPhTz)_2_[CuBr_4_] are identical to the samples cured with Co(Oct)_2_. E_a_ of the curing process EVE binder with MEKP and solution of the complex (TPhTz)_2_[CuBr_4_] reached 54.7 kJ/mol. Its result of 25.2 kJ/mol is higher than that of the standard curing system with Co(Oct)_2_. At the same time, the activation energy of MEKP decay during EVE curing without the participation of an accelerator is 85.23 kJ/mol. Thus, the solution of (TPhTz)_2_[CuBr_4_] in THF/DMSO accelerates the initiator decay process at room temperature, but for a longer time. The authors suggest that the curing mechanism may be accelerated by the appearance of free bromine in the system, which is consistent with previous studies.

The FGRP strength test revealed that the addition of (TPhTz)_2_[CuBr_4_] did not decrease flexural strength and hardness.

A new catalytic system has been developed for curing EVE, which can increase the lifetime of the binder during processing. Modern systems based on Co(Oct)_2_ have a narrow lifetime, which is inconvenient for the production of large composite structures using the VaRTM method. In this case, the addition of small amounts of an inhibitor (2,4-pentanedione) is required. Its use as a catalyst (TPhTz)_2_[CuBr_4_] 8 w.% can significantly increase the gel time without the addition of other components. This fact can also be useful for a factory with automatic systems for mixing and feeding components and binders into the mold, since the most modern mixing machines work with 2–3 components. Thus, the use of complex (TPhTz)_2_[CuBr_4_] allowed for the production of large polymer composite products with an extended injection time.

Further experiments could investigate the effect of different concentrations of complex (TPhTz)_2_[CuBr_4_] on the curing reaction and the mechanical properties of the resulting FGRP. Additionally, the use of other copper-based catalysts could also be explored to optimize the curing process and improve the mechanical properties of the composite material.

## Figures and Tables

**Table 1:** Crystallographic data and diffraction experiment conditions.

Empirical Formula	C_38_H_30_N_8_CuBr_4_	C_38_H_30_N_8_CuBr_3_
Formula weight (g/mol)	981.87	901.97
Temperature (K)	296(2)	150(2)
Crystal system	monoclinic	monoclinic
Space group	P 21/c	P 21/c
Cell Parameters	a = 12.0591(6) Å b = 15.2836(8) Å c = 20.8014(12) Å α = 90° β = 90.025(2)° γ = 90°	a = 16.2413(4) Å b = 12.9059(3) Å c = 17.6154(5) Å α = 90° β = 90.638(1)° γ = 90°
V (Å^3^)	3833.83(35)	3692.11(17)
Z	4	4
*ρ* calc (g/cm^–3^)	1.701	1.62255

**Table 2:** Basic interatomic distances (Å) and angles (°).

(TPhTz)_2_[Cu^II^Br_4_]						(TPhTz)_2_[Cu^I^Br_3_]					
Atom 1	Atom 2	d_1,2_ (Å)	Atom 3	d_1,3_ (Å)	Angle_2,1,3_ (°)	Atom 1	Atom 2	d_1,2_ (Å)	Atom 3	d_1,3_ (Å)	Angle_2,1,3_ (°)
Cu	Br(3)	2.3641(1)	Br(2)	2.3808(1)	100.304(3)	Cu	Br(2)	2.3753(1)	Br(3)	2.3815(0)	124.454(1)
Cu	Br(3)	2.3641(1)	Br(1)	2.3958(1)	101.526(2)	Cu	Br(2)	2.3753(1)	Br(1)	2.3817(0)	119.623(1)
Cu	Br(3)	2.3641(1)	Br(4)	2.4142(1)	132.481(2)	Cu	Br(3)	2.3815(0)	Br(1)	2.3817(0)	115.921(1)
Cu	Br(2)	2.3808(1)	Br(1)	2.3958(1)	123.233(2)	Cu	Br(1)	2.3817(0)	H(114)	2.7252(1)	80.798(1)
Cu	Br(2)	2.3808(1)	Br(4)	2.4142(1)	100.794(2)	Cu	Br(2)	2.3753(1)	H(114)	2.7252(1)	111.246(1)
Cu	Br(1)	2.3958(1)	Br(4)	2.4142(1)	101.533(2)	Cu	Br(3)	2.3815(0)	H(114)	2.7252(1)	77.525(1)

**Table 3:** FTIR spectroscopy data for (TPhTz)_2_[CuBr_4_], (TPhTz)_2_[CuBr_3_] and TPhTzCl.

Assignment	TPhTzCl (Figure S1)	(TPhTz)_2_[CuBr_4_] (Figure S2)	(TPhTz)_2_[CuBr_3_] (Figure S3)
n(H_3_O^+^)	3446 1709 1675	-	3439
n(C_arom_-H)	3057	3058	3043
R(Ph)	1608 1527	1607 1528	1607 1528
R(Tz)	1485 1455	1484 1457	1484 1455

**Table 4:** Solubility of complex (TPhTz)_2_[CuBr_4_] in different solvents.

Solvent	Complex Concentration, w.%
THF	6
DMSO	3
Styrene	7
THF + DMSO	8
Styrene + DMSO	5
CH_2_Cl_2_ + DMSO	7

**Table 5:** Gel and curing time of cobalt octoate with different concentrations and co-solvents.

Catalyst	Solvent	Gel Time, min	Curing Time, min
Co(Oct)_2_	-	16	38
	THF	22	40
	DMSO	37	55
	CH_2_Cl_2_	41	59
	Acetone	21	42
	THF + DMSO	19	46
	Styrene + DMSO	21	69
	CH_2_Cl_2_ + DMSO	16	56
(TPhTz)_2_[CuBr_4_]	THF	86	270
	DMSO	207	320
	Styrene	296	610
	THF + DMSO	66	144
	Styrene + DMSO	107	204
	CH_2_Cl_2_ + DMSO	32	98

**Table 6:** Shore D hardness of researched samples cured in the presence of 2% catalyst solution.

Catalyst	Solvent	Salt Concentration, w.%	Shore D Hardness	
			Before Heat Treatment	After Heat Treatment
Co(Oct)2	-	8	83	86
	THF		83.5	87
	DMSO		80	85
	CH2Cl2		83	87
	Acetone		83	84
	THF + DMSO		75	75.5
	Styrene + DMSO		78	82
	CH2Cl2 + DMSO		79	88.5
(TPhTz)2[CuBr4]	THF	6	80	79
	DMSO	3	76	83
	Styrene	7	74	82.5
	THF + DMSO	8	83	88
	Styrene + DMSO	5	75.5	87
	CH2Cl2 + DMSO	7	76	85

**Table 7:** FTIR spectroscopy data for cured Der /MEKP binder with different catalyst solutions.

Assignment	Catalyst Solution		
	Co(Oct)_2_ (Figure S5)	Co(Oct)_2_ with THF/DMSO (Figure S6)	(TPhTz)_2_[CuBr_4_] in THF/DMSO (Figure S7)
ν(OH) δ(OH)	3444 1377	3441 1387 1368	3448 1384 1363
ν(C-H)_Ar_ δ(C-H)_Ar_	3030 830	3058 829	3080 3027 828
ν(CH_3_)	2963 2929	2965 2931	2929
ν(CH_2_) δ(CH_2_)	2879 944	2875 944	2873 948
ν(COO) δ(COO)	1725 1181	1725 1181	1716 1710 1182
R(Ph)	1608 1507 1458	1644 1609 1510 1459	1653 1636 1607 1508 1457
ν(-C-O-C)	1297 1242 1115 1039	1296 1243 1112 1039	1297 1244 11201039
ν(-S-O-)	-	670	668

**Table 8:** Summary table of average strength values.

Catalyst	№	Heat Treatment	Flexural Strength, MPa	Shore D Hardness
Co(Oct)_2_	1	-	308	90
	2	120 °C	427	94
(TPhTz)_2_[CuBr_4_]	3	-	307	90.5
	4	120 °C	424	93.5

**Table 9:** The enthalpy value obtained from a DSC test of the samples.

Sample Containig Catalyst	Enthalpy Value, J/g
Co(Oct)_2_	6606
(TPhTz)_2_[CuBr_3_]	7477

**Table 10:** Binders curing kinetic parameters using the Kissinger approach.

Sample Containing Catalyst	Temperature of Peak (T), K	Heating Rate (v), K/min	ln(v/T^2^)	1000/T
(TPhTz)_2_[CuBr_3_]	354.7	2.5	−10.83	2.82
	369.2	5	−10.21	2.71
	379.6	10	−9.58	2.63
Co(Oct)_2_	332.4	2.5	−10.69	3.01
	345.6	5	−10.08	2.89
	370.2	10	−9.52	2.70
-	362.6	2.5	−10.87	2.75
	372.7	5	−10.25	2.65
	379.6	10	−9.57	2.64

**Table 11:** The Ea values.

Sample Containig Catalyst	tg(α)	Ea, kJ/mol
(TPhTz)_2_[CuBr_3_]	−6.5715	54.7
Co(Oct)_2_	−3.7196	31.0
-	−10.2390	85.23

## Data Availability

All related additional data are available in the Supplementary Materials. Samples of the compounds are available from the authors.

## Supplementary Materials

The following supporting information can be downloaded at: https://www.mdpi.com/article/10.3390/ijms241411808/s1.
