# Supplementary figures and images for: Coordination Compound (2,3,5-Triphenyltetrazolium)2[CuBr4] as Catalyst for the Curing Process of Epoxy Vinyl Ester Binders
Source: Int J Mol Sci. 2023 Jul 22;24(14):11808. doi: 10.3390/ijms241411808 (PMC10380813; doi:10.3390/ijms241411808)

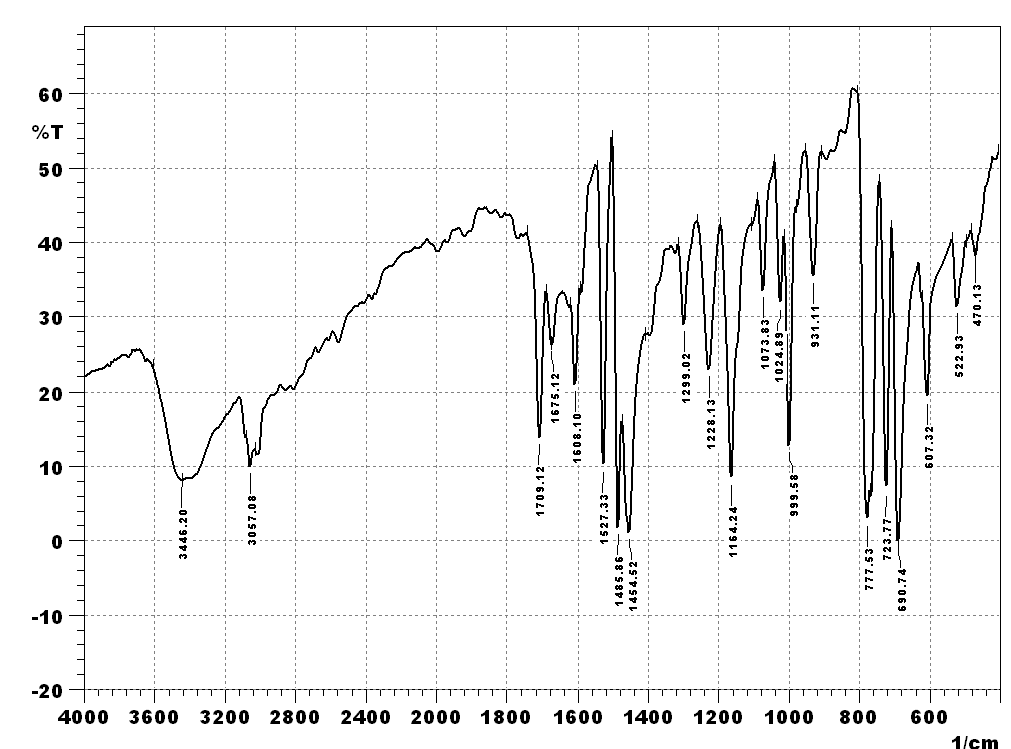

Supplement: Supplementary file 1 [file ijms-24-11808-s001.zip › Fig. S1 - FTIR of 2,3,5-TPhTz.bmp]

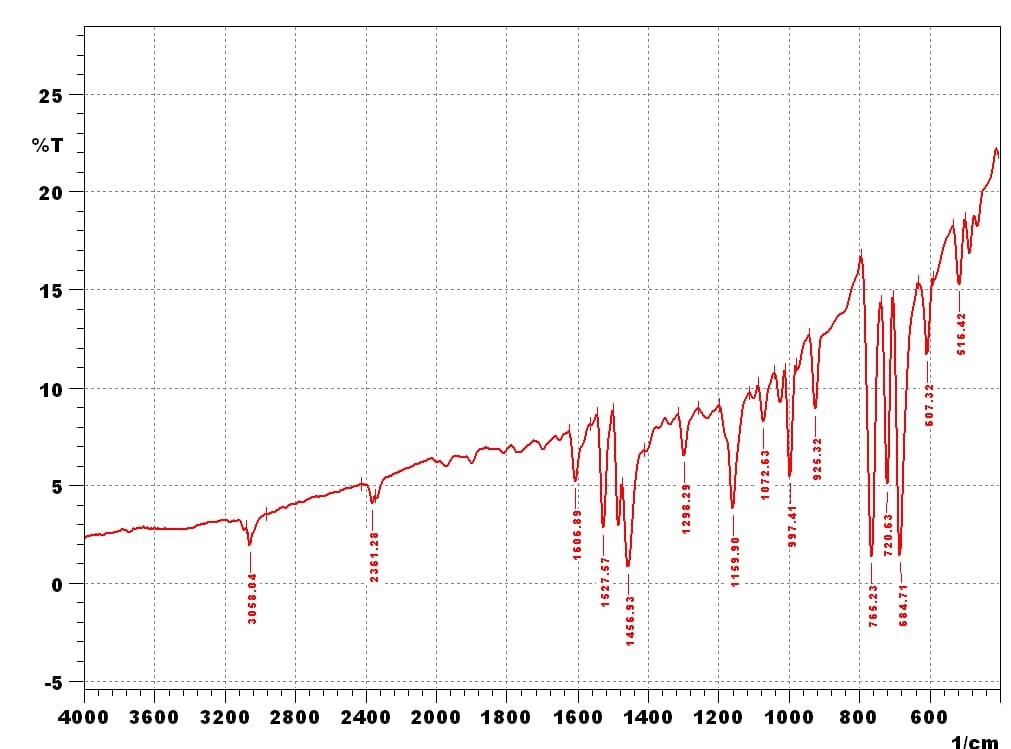

Supplement: Supplementary file 1 [file ijms-24-11808-s001.zip › Fig. S2 - FTIR of (2,3,5-TPhTz)2[CuBr4].bmp]

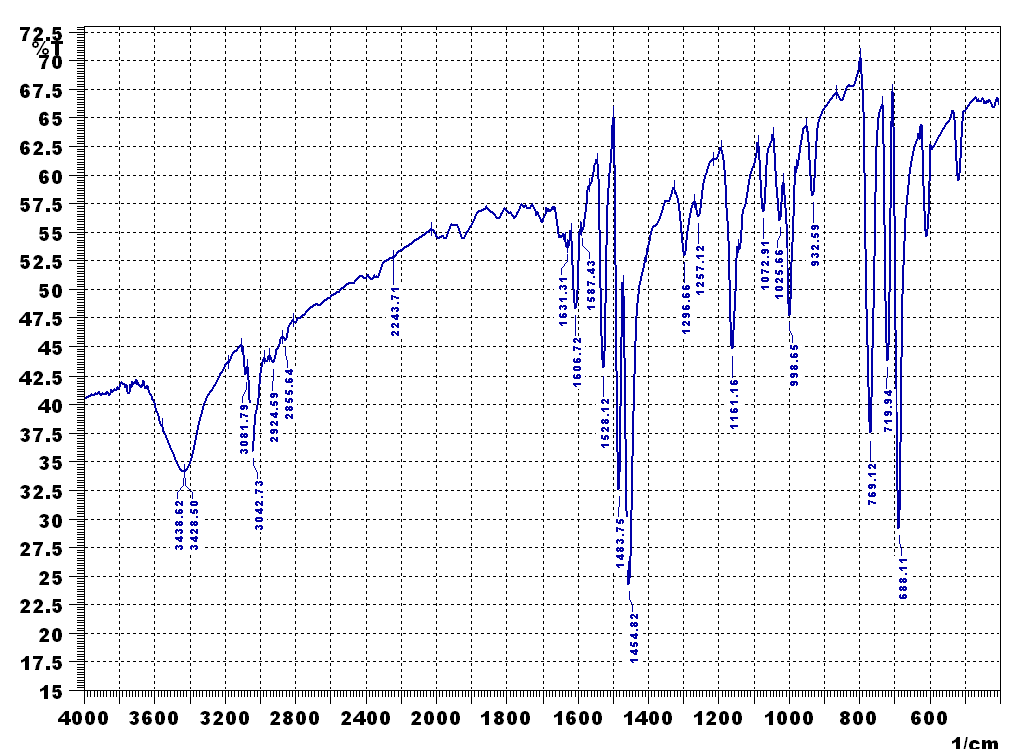

Supplement: Supplementary file 1 [file ijms-24-11808-s001.zip › Fig. S3 - FTIR of (2,3,5-TPhTz)2[CuBr3].bmp]

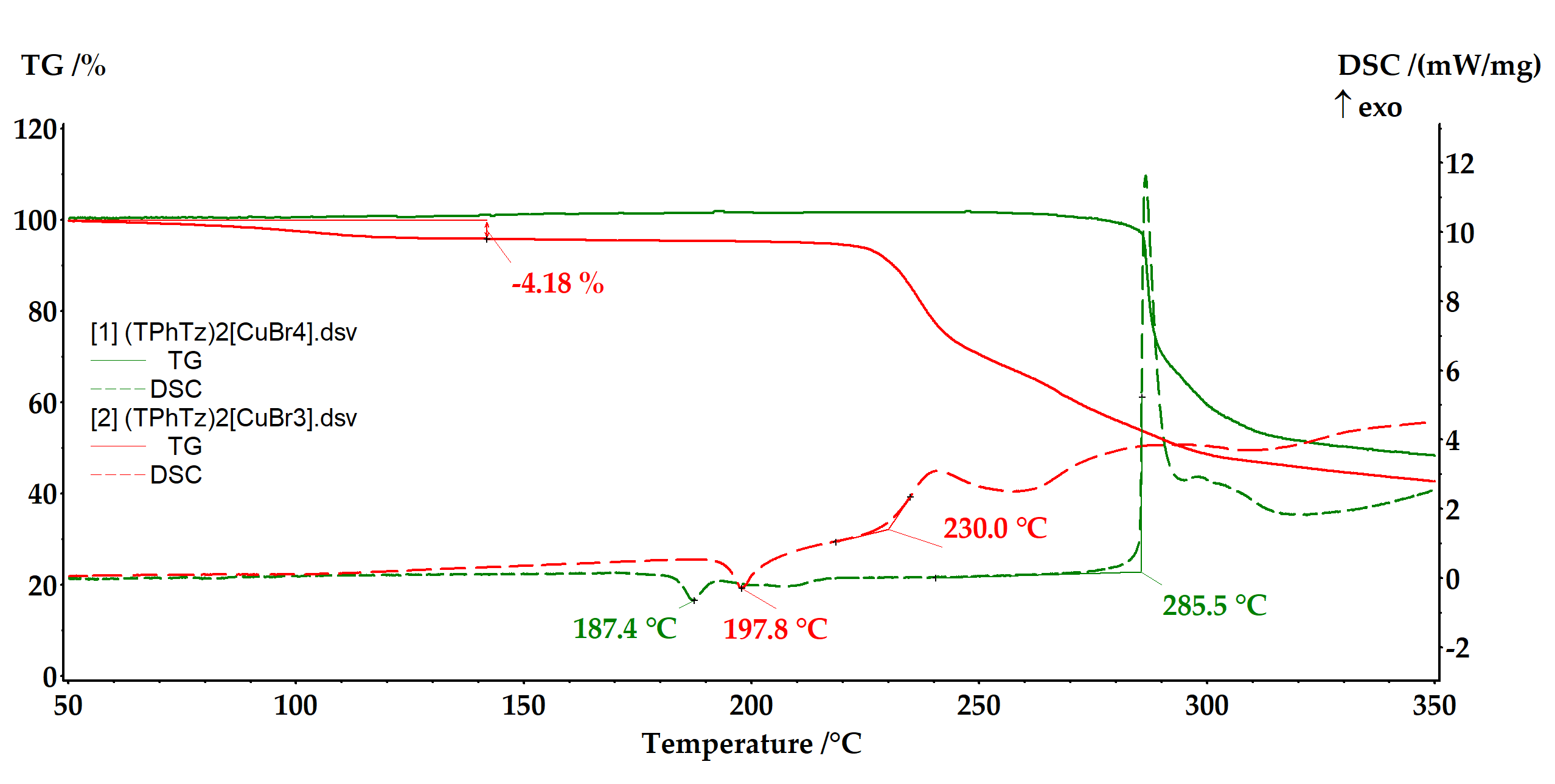

Supplement: Supplementary file 1 [file ijms-24-11808-s001.zip › Fig. S4 - Thermal Analysis of complexes (2,3,5-TPhTz)2[CuBr4] and (2,3,5-TPhTz)2[CuBr3].tif]

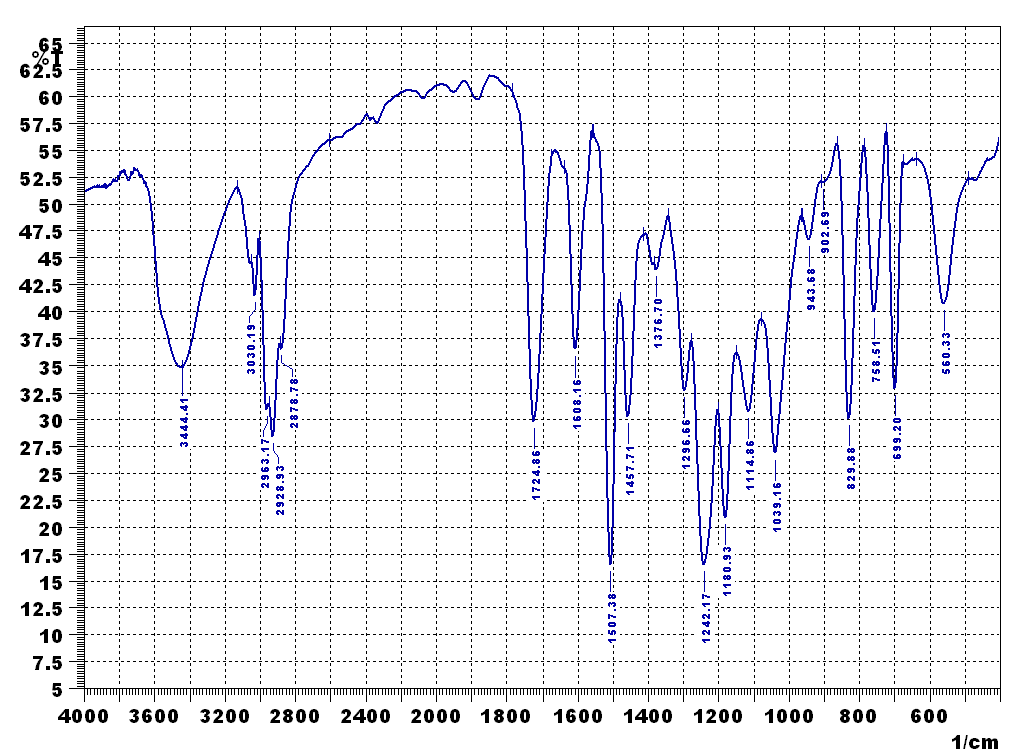

Supplement: Supplementary file 1 [file ijms-24-11808-s001.zip › Fig. S5 - FTIR of EVE with Co(Oct)2 and MEKP.bmp]

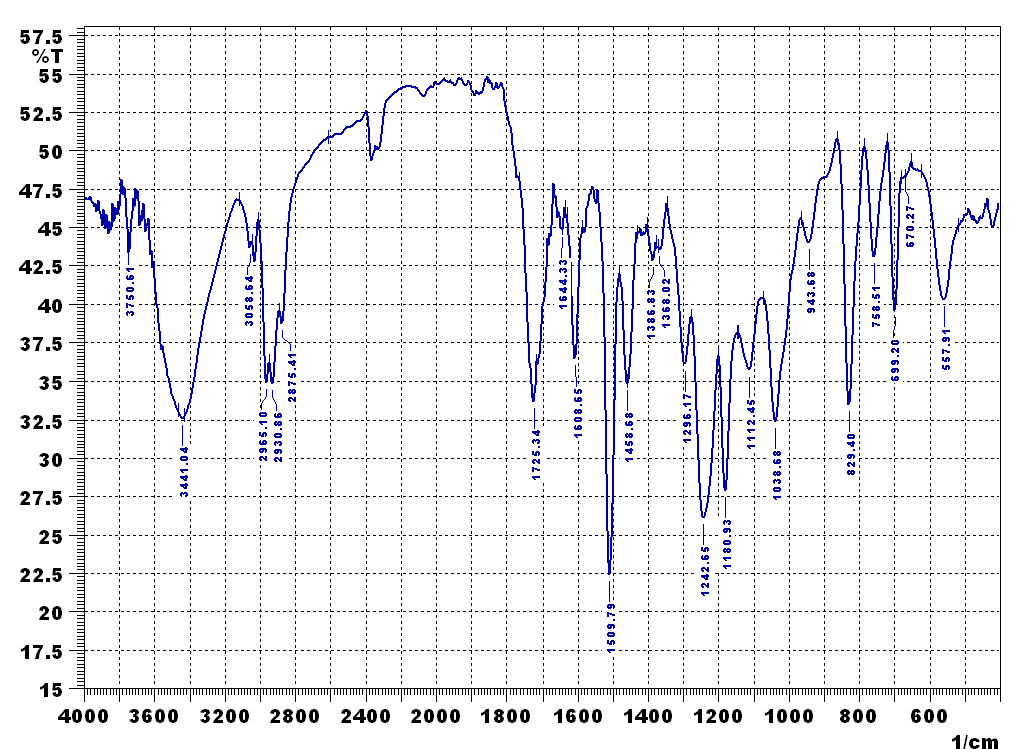

Supplement: Supplementary file 1 [file ijms-24-11808-s001.zip › Fig. S6 - FTIR of EVE with Co(Oct)2, THF+DMSO and MEKP.bmp]

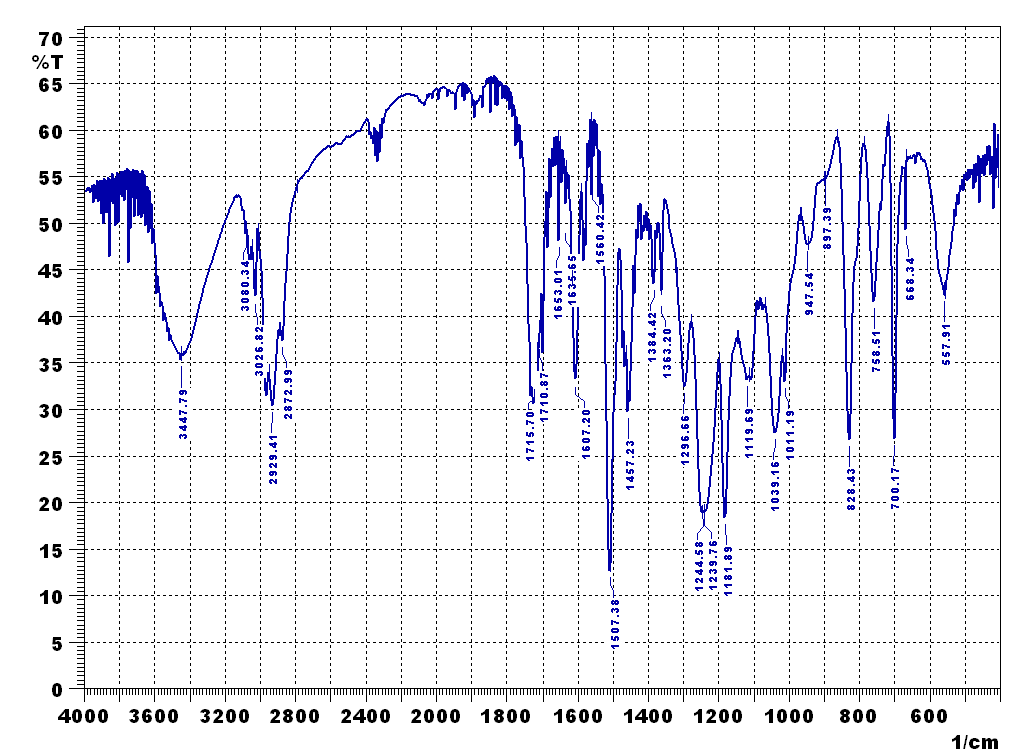

Supplement: Supplementary file 1 [file ijms-24-11808-s001.zip › Fig. S7 - FTIR of EVE with (TPhTz)2[CuBr4] in THF+DMSO and MEKP.bmp]

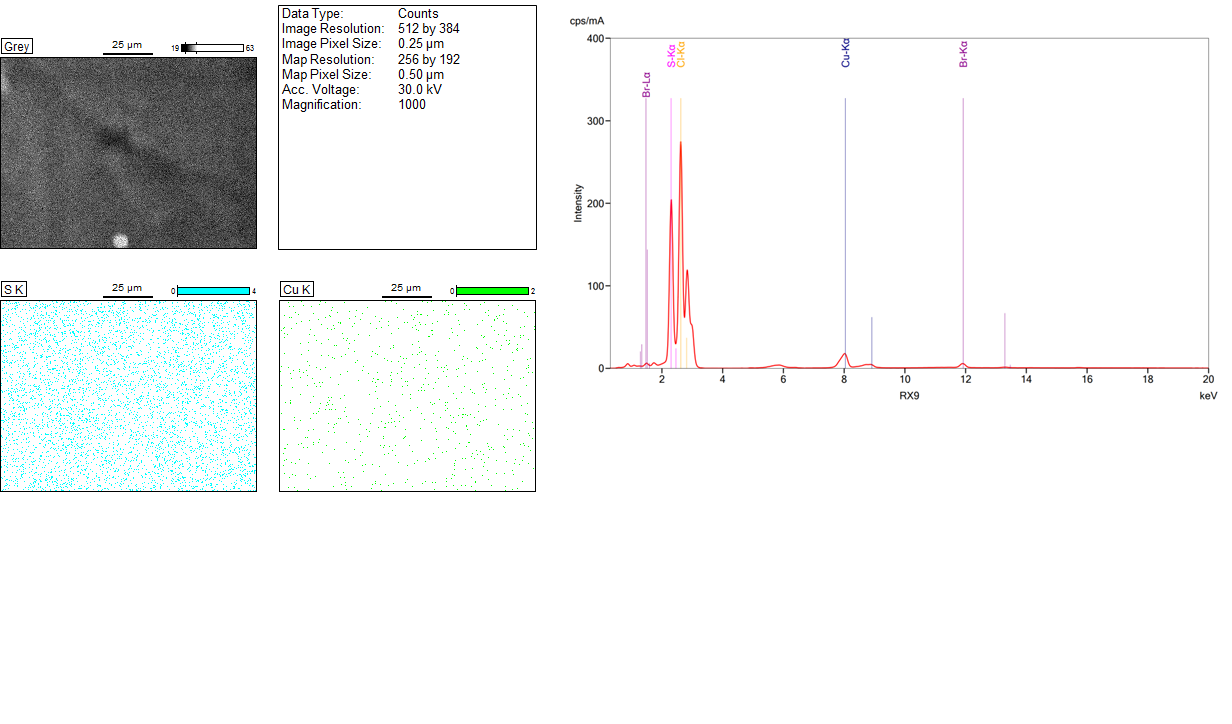

Supplement: Supplementary file 1 [file ijms-24-11808-s001.zip › Fig. S8 - EDX Analysis.tif]
